# Supplementary material for: A hidden cysteine in Fis1 targeted to prevent excessive mitochondrial fission and dysfunction under oxidative stress
Source: Nat Commun. 2025 May 6;16:4187. doi: 10.1038/s41467-025-59434-6 (PMC12056058; doi:10.1038/s41467-025-59434-6)
Supplement: Supplementary file 1 — Supplementary Information [file 41467_2025_59434_MOESM1_ESM.pdf]

Supplementary Information

A hidden cysteine in Fis1 targeted to prevent excessive mitochondrial fission and dysfunction under oxidative stress

Suman Pokhrel[1,2#], Gwangbeom Heo[1#], Irimpan Mathews[3], Shun Yokoi[2,4,5], Tsutomu Matsui[3], Ayori Mitsutake[5], Soichi Wakatsuki[2,4*], Daria Mochly-Rosen[1*]

[1]Department of Chemical and Systems Biology, Stanford University School of Medicine, Stanford, CA, USA
[2]Biological Sciences Division, SLAC National Accelerator Laboratory, Menlo Park, CA, USA
[3]Stanford Synchrotron Radiation Lightsource, Menlo Park, CA, USA
[4]Department of Structural Biology, Stanford University School of Medicine, Stanford, CA, USA
[5]Department of Physics, School of Science and Technology, Meiji University, Kanagawa, Japan

[#]These authors contributed equally

*Correspondence and requests for materials should be addressed to soichi.wakatsuki@stanford.edu or mochly@stanford.edu

	WT				pT34				pY38			
Res.	Ave. Phi	SD(Phi)	Ave. Psi	SD(Psi)	Ave. Phi	SD(Phi)	Ave. Psi	SD(Psi)	Ave. Phi	SD(Phi)	Ave. Psi	SD(Psi)
L15	-64.8	9.6	-34.1	10.5	-65.5	9.8	-34.4	10.6	-67.7	10.2	-33.4	11.2
K16	-68.5	10.1	-41.7	8.9	-68.1	9.9	-41.7	9.1	-67.1	10.6	-38.8	9.7
F17	-68.8	9.2	-35.4	8.1	-67.7	9.4	-36.2	8.6	-66.5	10.6	-40.5	10.5
E18	-62.7	8.2	-45.8	7.8	-62.6	8.3	-47.6	8.4	-60.5	8.9	-42.4	10.4
K19	-61.7	8.2	-39.1	8.6	-61.8	8.6	-38.2	9.3	-63.9	10.7	-36.5	11.5
K20	-63.9	8.4	-45.4	8.6	-64.3	12.2	-48.6	10.4	-67.6	15.6	-43.7	10.6
F21	-61.7	9.0	-48.6	7.8	-61.4	9.7	-45.9	9.3	-67.4	16.4	-38.0	13.8
Q22	-65.2	8.4	-35.7	9.2	-65.1	9.1	-36.3	10.4	-63.3	8.6	-36.1	10.0
S23	-68.3	9.3	-38.5	10.3	-68.8	9.7	-39.6	10.4	-69.1	10.4	-35.7	11.4
E24	-71.0	9.8	-37.3	10.2	-70.0	11.6	-36.0	14.1	-70.0	11.4	-38.0	23.6
L25	-64.7	8.9	-33.8	13.9	-66.5	12.3	-37.2	14.2	-65.9	11.2	-33.4	22.9

Supplementary Table 1 | Average φ and ψ values from the dihedral data sets in 30 independent 1.2 μs MD simulations for WT, pT34, and pY38.

	WT Dimer	WT	Thr34Asp	Tyr38Glu
Data collection parameters				
Instrument			SSRL BL4-2	
Type of Experiment			SEC-SAXS	
Beam Current (mA)			500	
Defining slits size (H mm × V mm)			0.3 × 0.30	
Detector distance (m)			1.1	
Detector			Pilatus3 X 1M	
Beam energy (keV)			11.0	
q range (Å^{-1})	0.0111-0.732	0.010–0.594	0.010–0.594	0.010–0.594
Sample cell			Quartz capillary (I*D*=~1.3mm)	
Temperature (K)			295	
Exposure time/frame (s)			1	
Frames per SEC-SAXS data set			500	
Number of blank images used for averaging			100	
Number of sample images used for averaging	15	5	5	5
Image numbers used for averaging	285-299	305-309	306-310	307-311
SEC column			Superdex 75 Increase 3.2/300	
HPLC flow rate (mL/min)			0.05	
Sample concentration (mg/ml)	5	6	6	6
SEC injection volume (µL)	100	30	30	30
Buffer			50 mM Tris/HCl pH 8.0 and 150 mM NaCl	
Software employed				
Primary data reduction			*SasTool/SECPipe*	
Data processing			*PRIMUS*	
P(r) analysis			*GNOM*	
Atomistic modeling			*CORAL*	
ab initio modeling			*Dammif, DENSS*	
Structural parameters				
Guinier analysis				
$I(0)$	0.013 ± 0.000058	0.017 ± 0.000059	0.023 ± 0.000063	0.015 ± 0.000054
R_g (Å)	25.36 ± 0.17	17.91 ± 0.10	17.55 ± 0.08	17.12 ± 0.10
q_{min} (Å^{-1})	0.0111	0.0172	0.0172	0.0149
qR_g range	0.28 – 1.27	0.31 – 1.30	0.30 - 1.30	0.26 - 1.29

Supplementary Table 2 | SAXS data collection and analysis (contd.).

	WT Dimer	WT	Thr34ASP	Tyr38Glu
Data collection parameters				
$I(0)$, Guinier	0.013	0.017	0.023	0.015
R_g (Å), Guinier	25.43	18.18	17.75	17.11
$I(0)$, $P(r)$	0.013	1.006	1.372	0.885
R_g (Å), $P(r)$	25.44	18.20	17.76	17.11
D_{max} (Å)	83.00	61.00	60.00	57.00
q range (Å^{-1})	0.11-0.32	0.017 – 0.45	0.017 – 0.45	0.015 – 0.45
Porod volume estimate (Å^3)	42619	24500	25800	28600

Supplementary Table 2 | SAXS data collection and analysis.

System	Replicas	Time length for each simulation [μs]	Box dimensions [Å]	Total number of atoms	Total number of water molecules	Salt concentration [mM]
**WT**	30	1.2	86.8×76.6×72.2	38376	12082	150
**pT34**	30	1.2	86.8×76.6×72.2	38376	12081	150
**pY38**	30	1.2	86.8×76.6×72.2	38376	12081	150

Supplementary Table 3 | MD simulations overview.

	Fis1-WT	Fis1-T34D	Fis1-T34E
**Crystallographic parameters**			
Space group	$P6_5$	$P4_12_12$	$P4_12_12$
Unit-cell dimensions	58.33, 58.33, 156.29Å<br>90, 90,90˚	43.51, 43.51, 120.79Å<br>90, 90,90˚	43.25, 43.25, 119.81Å<br>90, 90, 90˚
**Data collection statistics**			
Resolution limits (outer shell) (Å)	30.9-1.95(2.00-1.95)	35.3-2.51 (2.58-2.51)	35.1-2.37 (2.43-2.37)
No: of observed reflections (outer shell)	297793 (21658)	71168 (5519)	53843 (3928)
No: of unique reflections (outer shell)	21571 (1587)	4385 (327)	5063 (357)
Completeness (outer shell)	98.6 (98.3)	100 (100)	100 (100)
CC1/2 (outer shell)	99.9 (66.3)	99.8 (82.5)	100 (65.4)
R_{sym}[a] (outer shell) (%)	9.2 (254.3)	9.8 (187.3)	6.3 (213.6)
Mean I/σ(I) (outer shell)	18.6 (1.7)	14.3 (1.5)	18.7 (1.5)
**Refinement statistics**			
Resolution limits (Å)	30.9-1.95	35.3-2.51	35.1-2.37
Number of reflections (%)	21564 (98.6)	4380 (99.9)	5059 (99.9)
Reflections used for R_{free}	1079	264	304
R_{factor}[b] (%)	18.5	23.4	23.9
R_{free} (%)	23.1	28.8	29.1
Model contents (average B($Å^2$))			
Protein atoms	2044 (43.3)	828 (86.5)	823 (84.2)
Ions	2 (57.6)	0	0
Water molecules	147 (50.7)	16 (88.2)	19 (79.8)
RMS deviations			
Bond length (Å)	0.007	0.009	0.009
Bond angle (°)	0.96	1.09	1.09
Ramachandran (favored (%)/outliers)	99/0	95/0	97/0

[a] $R_{sym} = \Sigma|I_{avg}-I_i|/\Sigma I_i$

[b] R factor = $\Sigma|F_p - F_{pcalc}|/\Sigma F_p$, where F_p and F_{pcalc} are the observed and calculated structure factors; R_{free} is calculated with 5% of the data.

Supplementary Table 4 | Crystallographic parameters, data collection and refinement statistics (contd.).

	Fis1-Y38E	Fis1-Y38E (Move)	Fis1-SP11
**Crystallographic parameters**			
Space group	$P2_1$	$P2_12_12$	$P2_12_12_1$
Unit-cell dimensions	43.47, 43.07, 61.24Å<br>90.0, 99.3,90.0˚	68.50, 81.44, 47.19Å<br>90, 90, 90˚	43.17, 51.79, 59.99Å<br>90˚, 90˚, 90˚
**Data collection statistics**			
Resolution limits (outer shell) (Å)	38.0-2.09(2.14-2.09)	38.86-1.53 (1.57-1.53)	35.04-1.9 (1.95-1.90)
No: of observed reflections (outer shell)	89059 (6459)	516173 (39742)	129790 (9777)
No: of unique reflections (outer shell)	13234 (967)	40271 (2961)	11085 (802)
Completeness (outer shell)	98.2 (97.2)	99.2 (99.5)	100 (100)
CC1/2 (outer shell)	99.9 (60.5)	100 (75.0)	99.7 (69.2)
R_{sym}[a] (outer shell) (%)	5.2 (156.5)	6.3 (246.7)	8.3 (176.7)
Mean I/σ(I) (outer shell)	18.8 (1.7)	22.3 (1.8)	14.8 (1.5)
**Refinement statistics**			
Resolution limits (Å)	38.0-2.09	38.86-1.53	35.04-1.90
Number of reflections (%)	13227 (98.2)	38258 (99.2)	11078 (99.9)
Reflections used for R_{free}	661	2014	554
R_{factor}[b] (%)	19.0	18.1	21.1
R_{free} (%)	24.4	22.6	25.4
Model contents (average B(Å^2))			
Protein atoms	1634 (61.0)	2184 (30.6)	830 (62.8)
Ion/Ligand	0	24/42.1	22/75.1
Water molecules	82 (64.7)	198 (41.7)	50 (71.1)
RMS deviations			
Bond length (Å)	0.007	0.004	0.007
Bond angle (°)	0.90	1.14	1.03
Ramachandran (favored (%)/outliers)	99/0	100/0	100/0

[a] $R_{sym} = \Sigma|I_{avg}-I_i|/\Sigma I_i$

[b] R factor = $\Sigma|F_p - F_{pcalc}|/\Sigma F_p$, where F_p and F_{pcalc} are the observed and calculated structure factors; R_{free} is calculated with 5% of the data.

Supplementary Table 4 | Crystallographic parameters, data collection and refinement statistics.

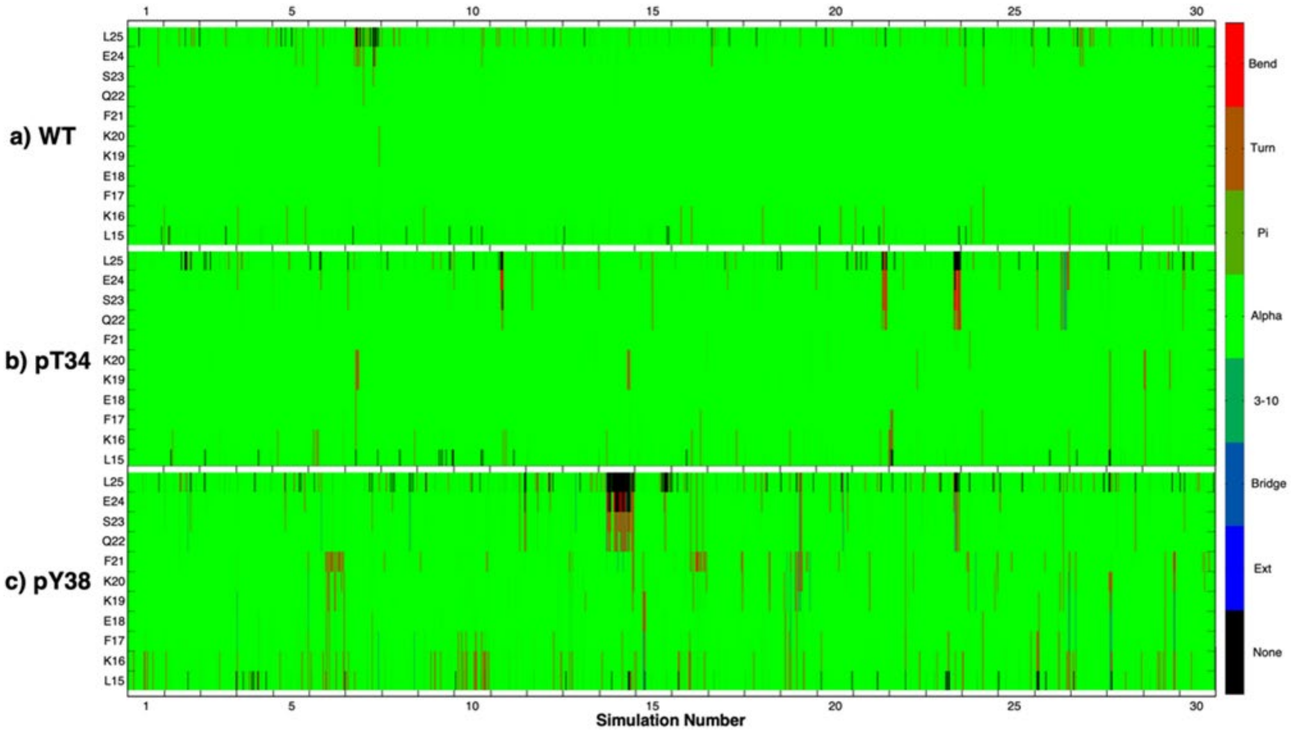

Supplementary Fig. 1 | Secondary structural propensities of residues in α1 helix (aa 15-25) from 30 independent 1.2 μs MD simulations for WT, pT34, and pY38.

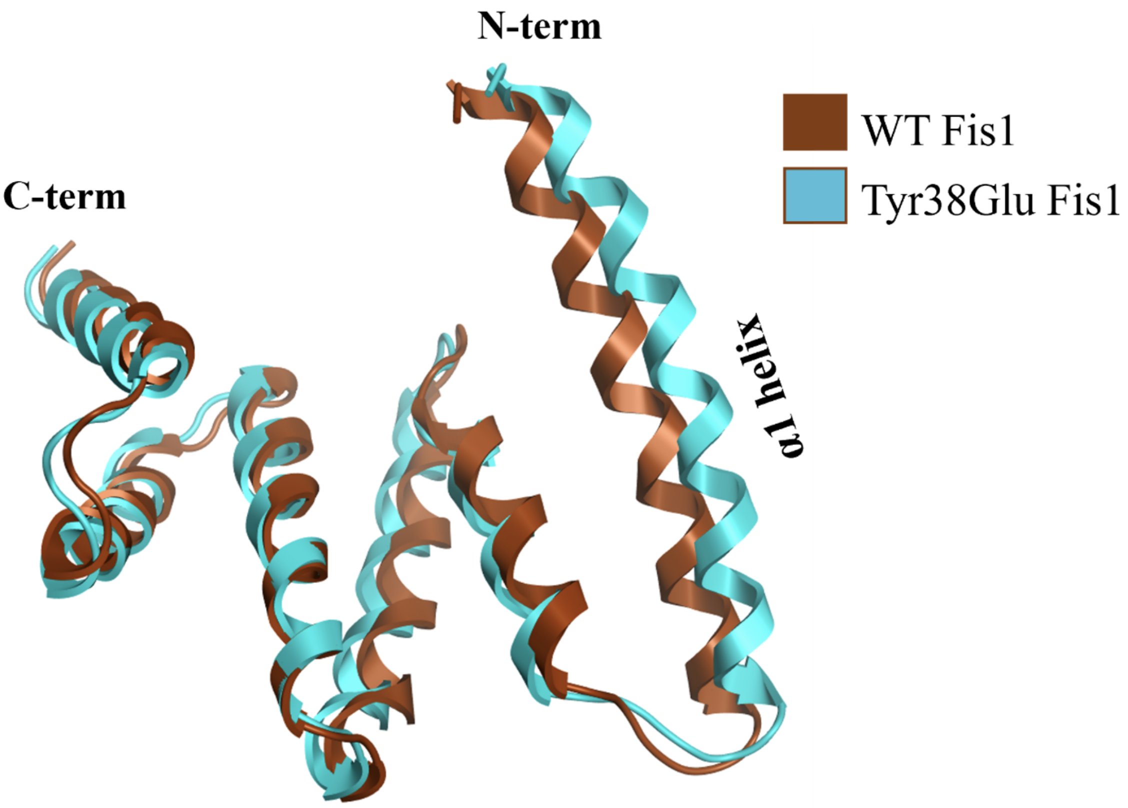

Supplementary Fig. 2 | Overlay of WT Fis1(brown) and Tyr38Glu Fis1(cyan). N-terminus of Tyr38Glu is intact but shifted.

	WT	T34D	Y38E
D_{max} -5Å	24310	25452	28421
D_{max}	24508	25794	28630
D_{max} + 5Å	24531	25795	28714
Average Volume	24450	25680	28588
SD	121	198	151
% of SD	0.50	0.77	0.53

Supplementary Fig. 3a | Porod Volumes with different Dmax values (+/- 5Å) using Primus. The q-range used for this analysis was fixed ($0.016 < q < 0.45$) for comparison.

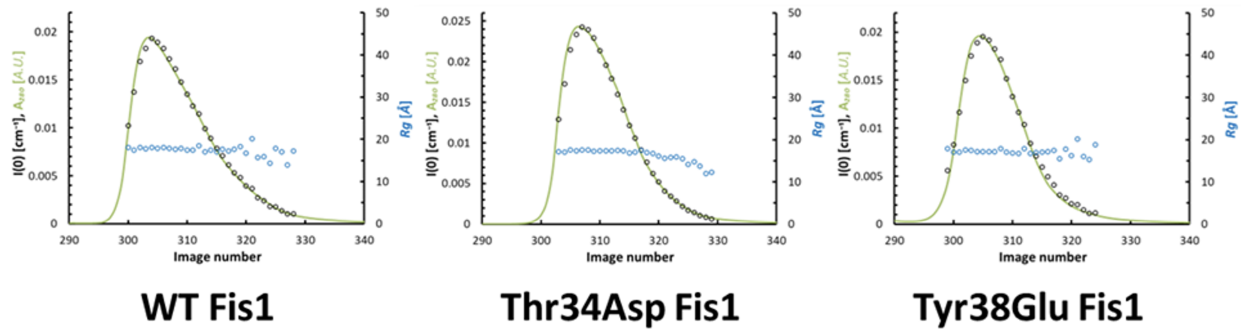

Supplementary Fig. 3b | Image number vs R_g and I(0) plots for WT and phosphorylation mimic mutants of Fis1.

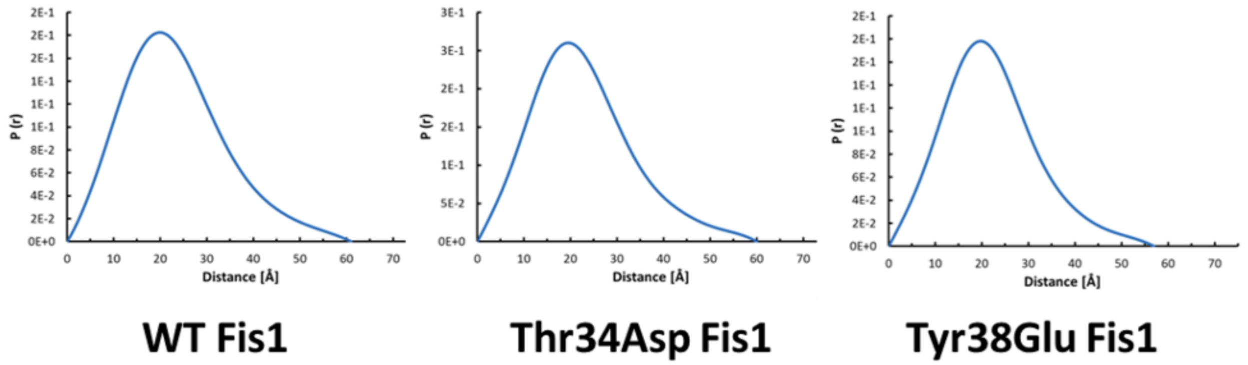

Supplementary Fig. 3c | P(r) plots for WT and phosphorylation mimic mutants of Fis1.

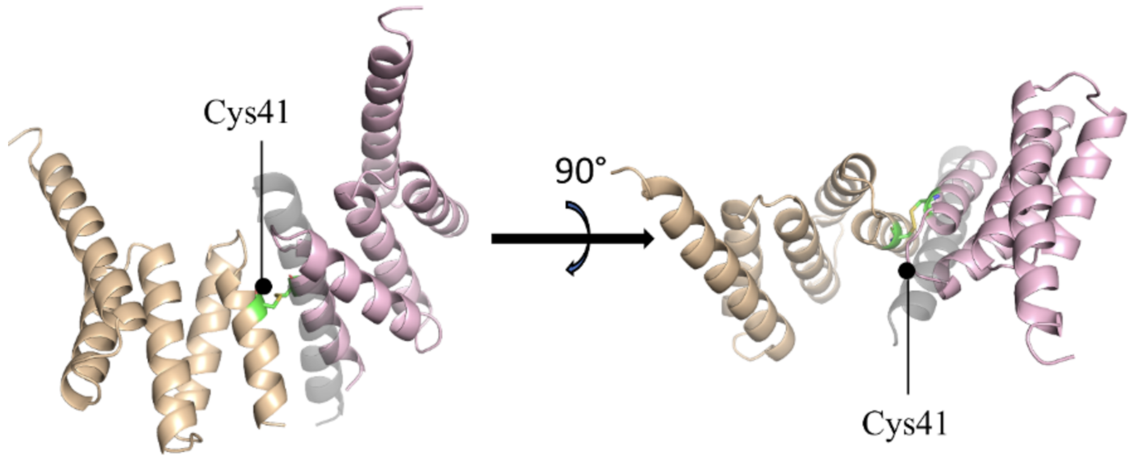

Supplementary Fig. 4 | Overlay of Thr34Asp covalent dimer and WT Fis 1 structure. WT Fis1with intact N-terminus (transparent grey) overlayed with Thr34Asp Fis1 covalent dimer (wheat and pink

monomers) and Cys41 shown in green. α1 helix of the WT Fis1 occupies same space as the α2 helix of another monomer suggesting unlikelihood of Cys41 covalent dimerization in WT Fis1 with intact α1 helix.

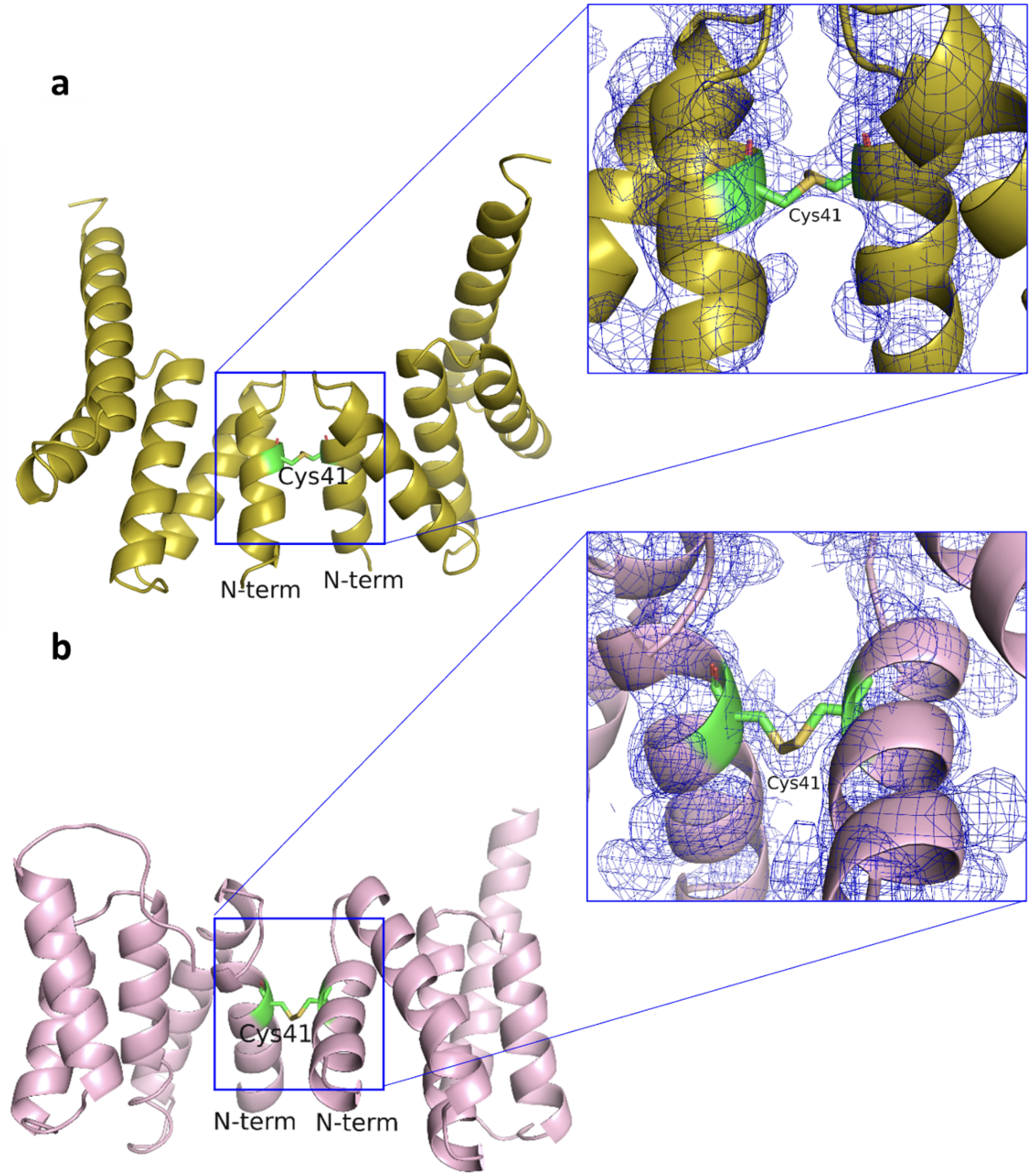

Supplementary Fig. 5 | Crystal structures of Thr34Glu and Tyr38Glu Fis1 covalent dimer. Covalent dimer structures of Thr34Glu Fis1 (**a**) and Tyr38Glu Fis1 (**b**). Continuous electron density (Composite omit map (σ = 1.0)) of the disulfide bonds between two monomers is highlighted.

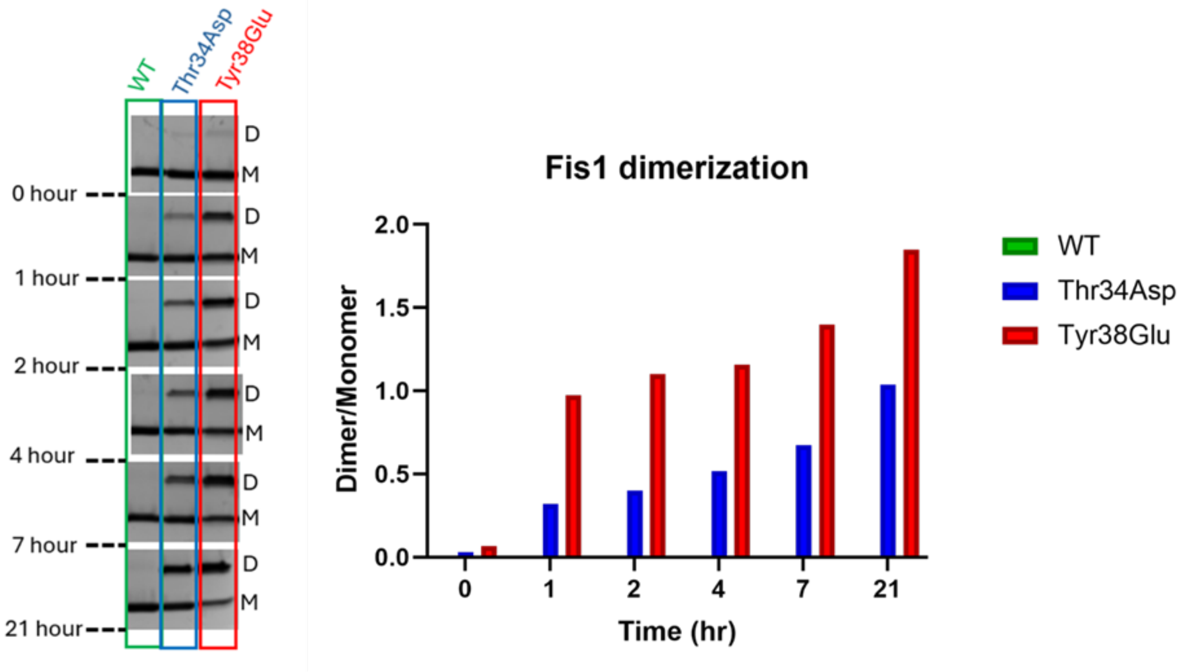

Supplementary Fig. 6 | Non-reducing non-heat denatured SDS-PAGE showing monomers and dimers of different constructs of Fis1 immobilized to HisPur^{TM} Ni-NTA magnetic beads at different time points. Equal amount of beads initially loaded with about 6 µg protein were loaded in each lane. Uncropped images are provided in the Source Data file.

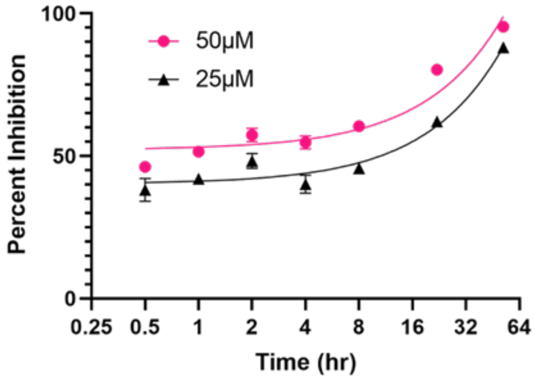

Supplementary Fig. 7 | Inhibition of CPM fluorescence by SP11 in Thr34Asp Fis1 at different time points. 4 µM protein and 8 µM CPM were used in the assay. Each data point represents mean and error bar represents standard deviation of 4 technical replicates.

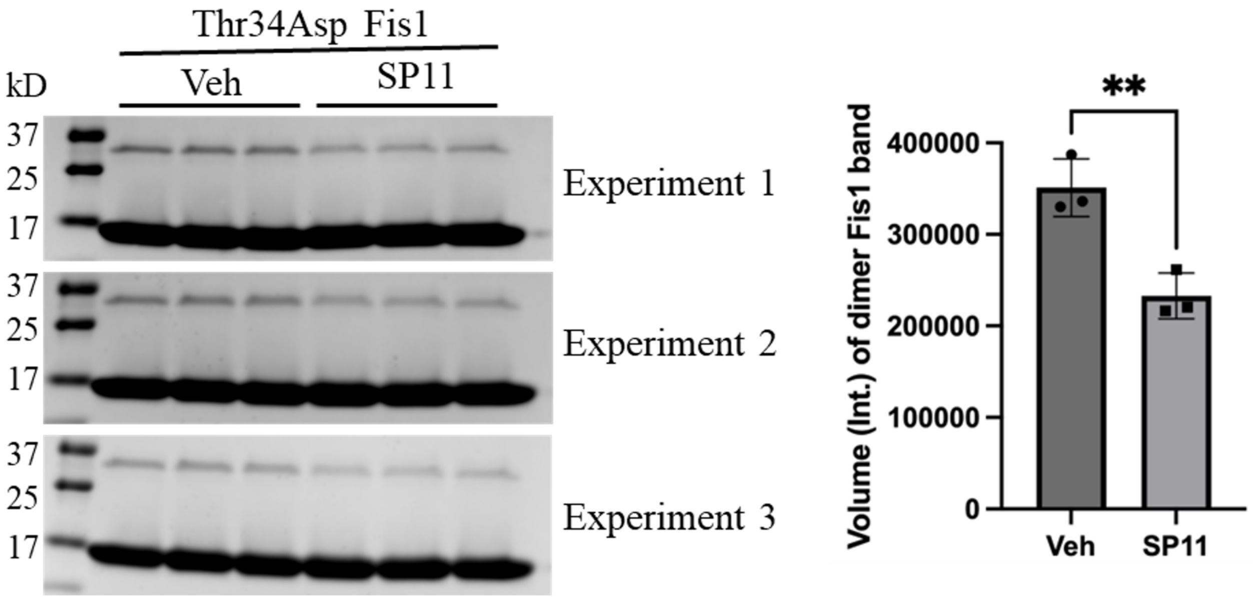

Supplementary Fig. 8 | Non-reducing non-heat denatured SDS-PAGE showing inhibition of dimerization by SP11 treatment and quantification of dimer bands. Equal amount of beads initially loaded with 20 µg protein (53% of total initial beads; about 10.6 µg protein) were loaded in each lane. Plotted value represents mean of 3 independent experiments each with 3 technical replicates. Error bar represents standard deviation. Two tailed unpaired t-test was performed. P-value results indicated by stars (** $p < 0.01$). Source data including all statistics (degrees of freedom, p values, effect sizes, and confidence intervals) are provided in the Source Data file.

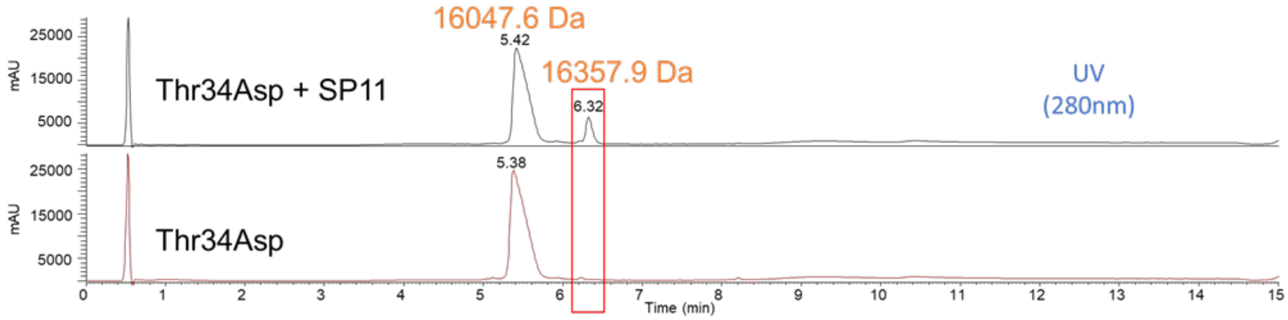

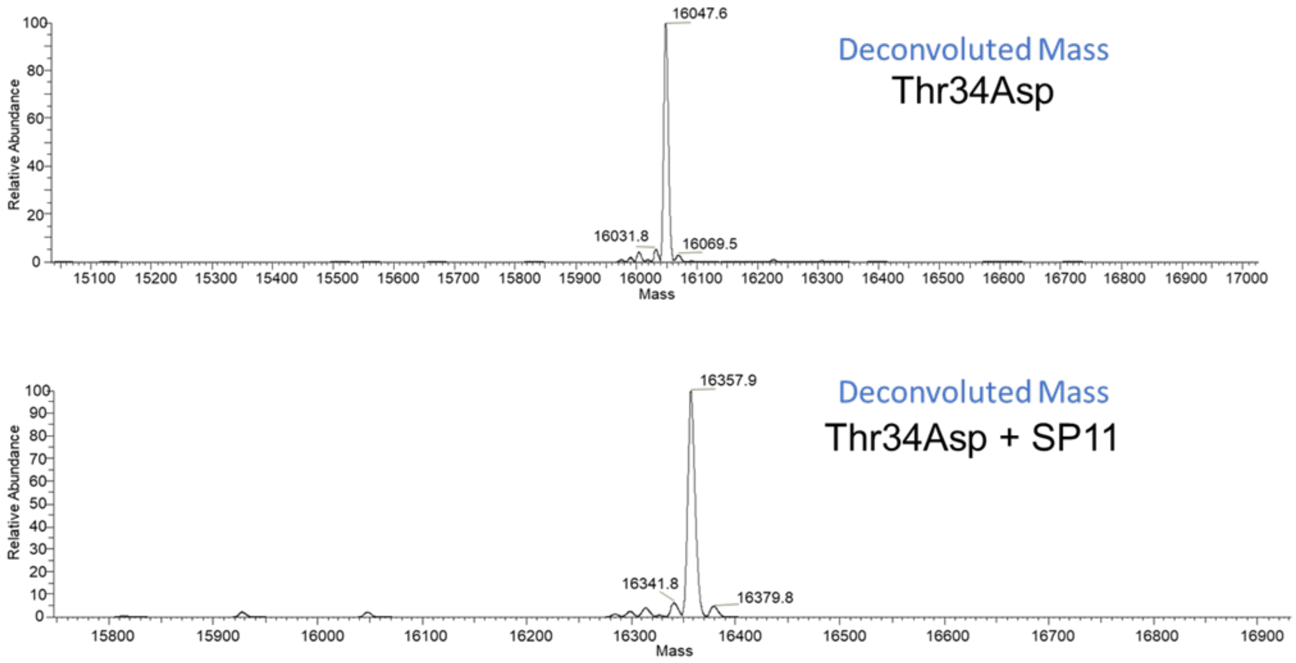

Supplementary Fig. 9 | Intact mass spectra from the same sample used in peptide mapping. Thr34Asp Fis1 + SP11 sample shows a new peak shifted by about 310 Da (weight of SP11 adduct) compared to untreated Thr34Asp Fis1.

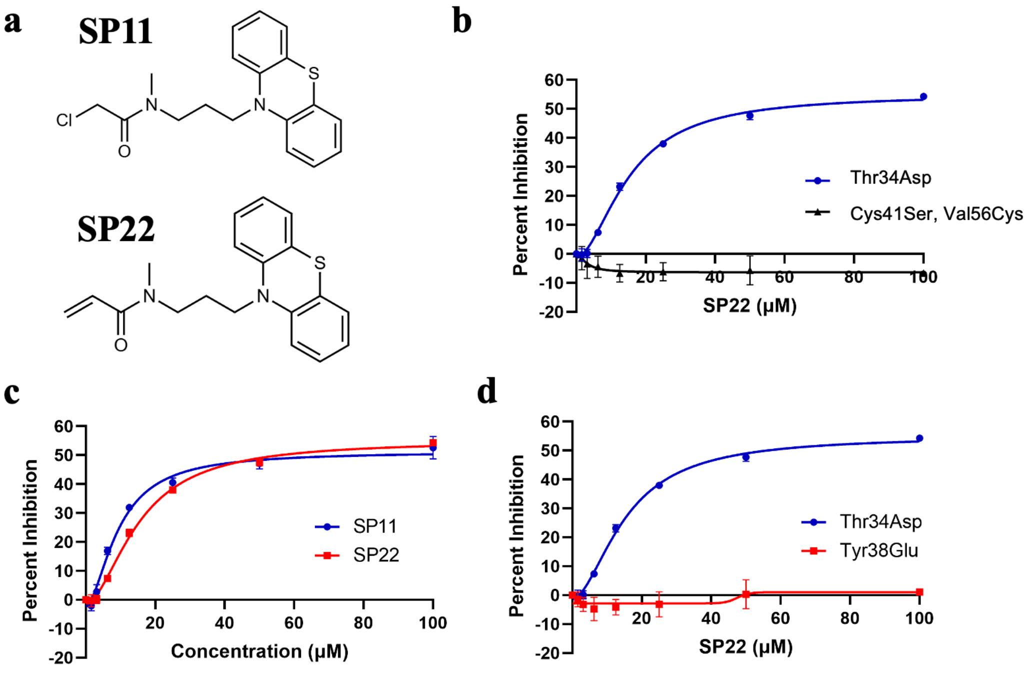

Supplementary Fig. 10 | Inhibitory properties of SP11 and SP22. a, Structure of SP11 and SP22. **b**, Inhibition of CPM fluorescence by SP22 in Thr34Asp Fis1 but not in counter screen construct (Cys41Ser, Val56Cys Fis1 double mutant). **c,** SP22 inhibits Thr34Asp Fis1 with slightly lower apparent IC50 compared to SP11. **d**, SP22 binds to Cys41 in Thr34Asp Fis1 mutant but doesn't bind to Cys41 when Tyr38 is mutated. 4 µM protein and 8 µM CPM were used in the assays. Each data point represents mean of 3 independent experiments each with 3 technical replicates. Error bar represents standard deviation.

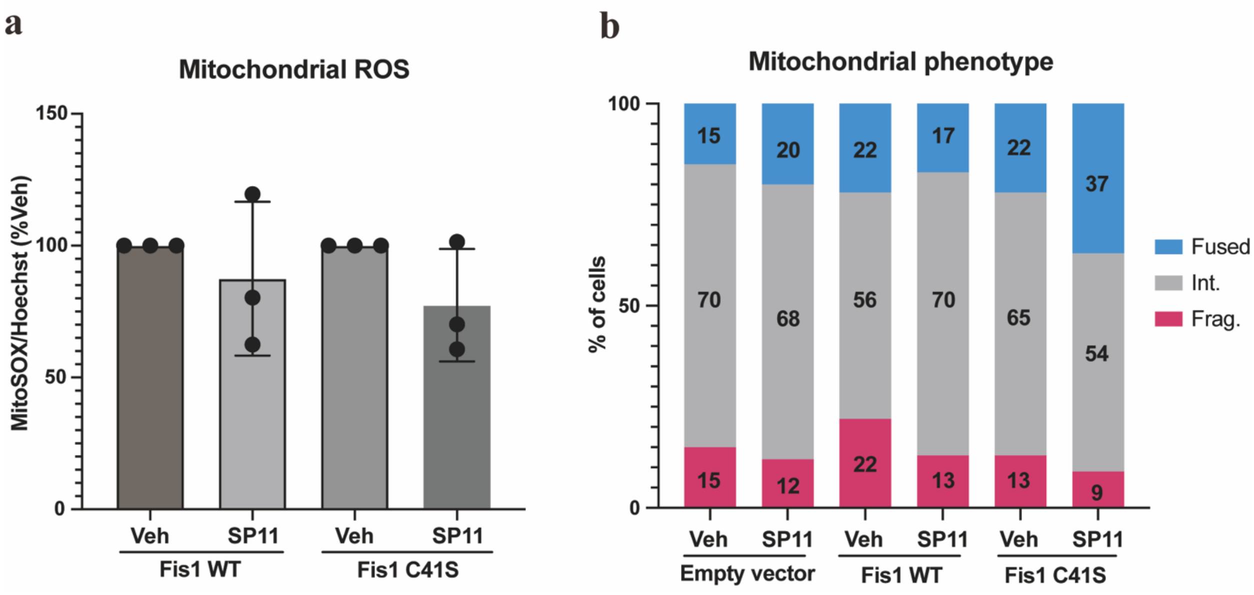

Supplementary Fig. 11 | Effect of SP11 on non-stimulated cells. a, Be(2)-M17 WT and Cys41Ser cells were treated with DMSO or SP11 (250 nM) for 24h. Mitochondrial ROS was measured using MitoSOX^{TM} mitochondrial superoxide indicator. MitoSOX^{TM} signal was normalized to Hoechst 33342. Each data point represents the mean of an independent experiment with 6 replicates each (n= 3). **b,** Fis1 knock-out MEFs were transfected with empty vector, Fis1 WT or Fis1 C41S plasmid for 24h. The cells were treated with vehicle control (DMSO) or SP11 (250 nM) for 6.5h. Mitochondrial morphology in live cells was analyzed using the same method as HK-2 cells. Average mitochondrial area per cell was normalized to vehicle mean of the empty vector-transfected vehicle-treated cells. Cells from were categorized into three groups with using the same method as HK-2 cells. The original violin plot is presented in Supplementary Fig. 12. n= 121 cells for empty vector Veh; 94 cells for empty vector SP11; 101 cells for Fis1 WT Veh; 86 cells for Fis1 WT SP11; 122 cells for Fis1 C41S Veh; 119 cells for Fis1 C41S SP11. WT, wild-type; Veh, vehicle; Int., intermediate; Frag., fragmented.

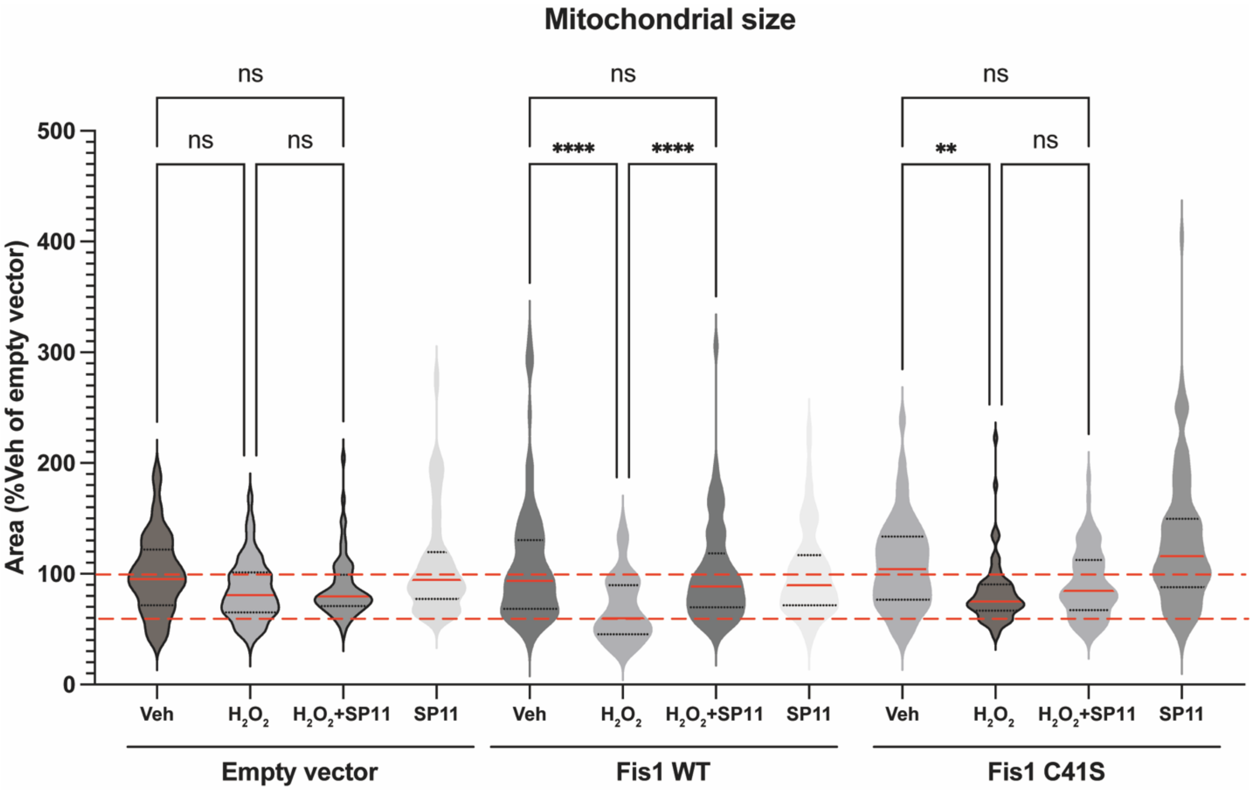

Supplementary Fig. 12 | Mitochondrial morphology in MEFs. Fis1 knock-out MEFs were transfected with empty vector, Fis1 WT or Fis1 C41S plasmid for 24h. The cells were pre-treated with vehicle control or SP11 (250 nM) for 30 min, then treated with vehicle control or H_2O_2 (50 μM) for additional 6h. Mitochondrial morphology in live cells was analyzed using the same method as HK-2 cells. Average mitochondrial area per cell was normalized to vehicle mean of the empty vector-transfected vehicle-treated cells. (Center line, median; upper and lower dotted lines, quartiles) n= 121 cells for empty vector Veh; 110 cells for EV H_2O_2; 66 cells for EV H_2O_2+SP11; 94 cells for EV SP11; 101 cells for WT Veh; 86 cells for WT H_2O_2; 74 cells for WT H_2O_2+SP11; 86 cells for WT SP11; 122 cells for C41S Veh; 74 cells for C41S H_2O_2; 87 cells for C41S H_2O_2+SP11; 119 cells for C41S SP11. P-value results indicated by stars (ns $p \geq 0.05$, ** $p < 0.01$, **** $p < 0.0001$). WT, wild-type; Veh, vehicle.

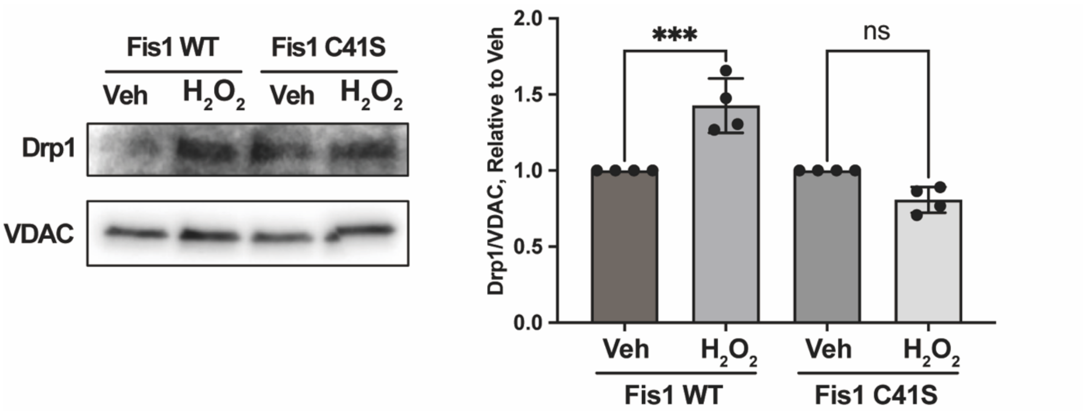

Supplementary Fig. 13 | Mitochondrial translocation of Drp1 in Fis1 WT and Fis1 C41S-transfected MEFs. Fis1 knock-out MEFs were transfected with Fis1 WT or Fis1 C41S plasmid for 24h. The cells were treated with vehicle or H_2O_2 (50 μM) for 24h. Mitochondrial fraction was prepared using the same method as HK-2 cells. Densitometry graph was obtained from four independent experiments. P-value results indicated by stars (ns $p \geq 0.05$, *** $p < 0.001$). Veh, vehicle; WT, wild-type.

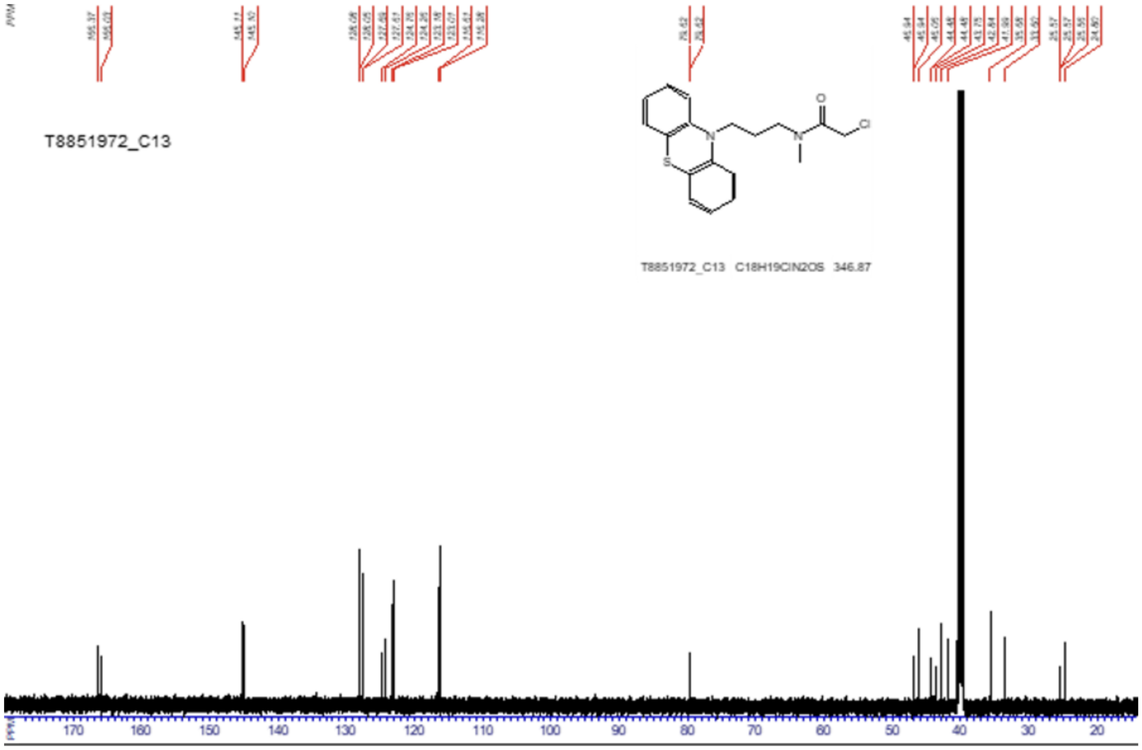

Supplementary Fig. 14a| SP11 ^{13}C NMR spectrum.

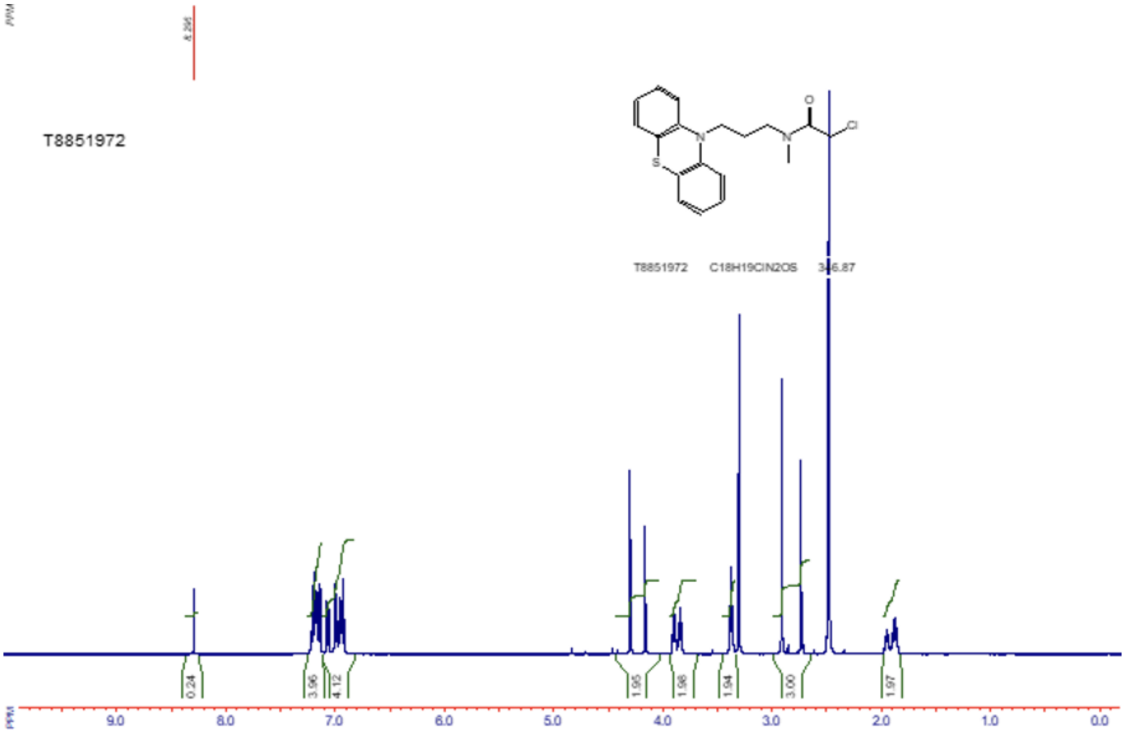

Supplementary Fig. 14b | SP11 ^{1}H NMR spectrum.

Compound Table

Label	Tgt Score	Mass Error (ppm)	Tgt Formula	Obs. RT	Ref. Mass	Obs. Mass
Cpd 1: C18 H19 Cl N2 O S; 3.052	93.14	-0.26	C18 H19 Cl N2 O S	3.052	346.09066	346.09057

Obs. *m/z*	Obs. RT	Obs. Mass	Tgt Formula	Tgt Mass	Tgt Mass Error (ppm)	RT Diff.	Find Cpds Algorithm
347.09806	3.052	346.09057	C18 H19 Cl N2 O S	346.09066	-0.26	Find By Formula	

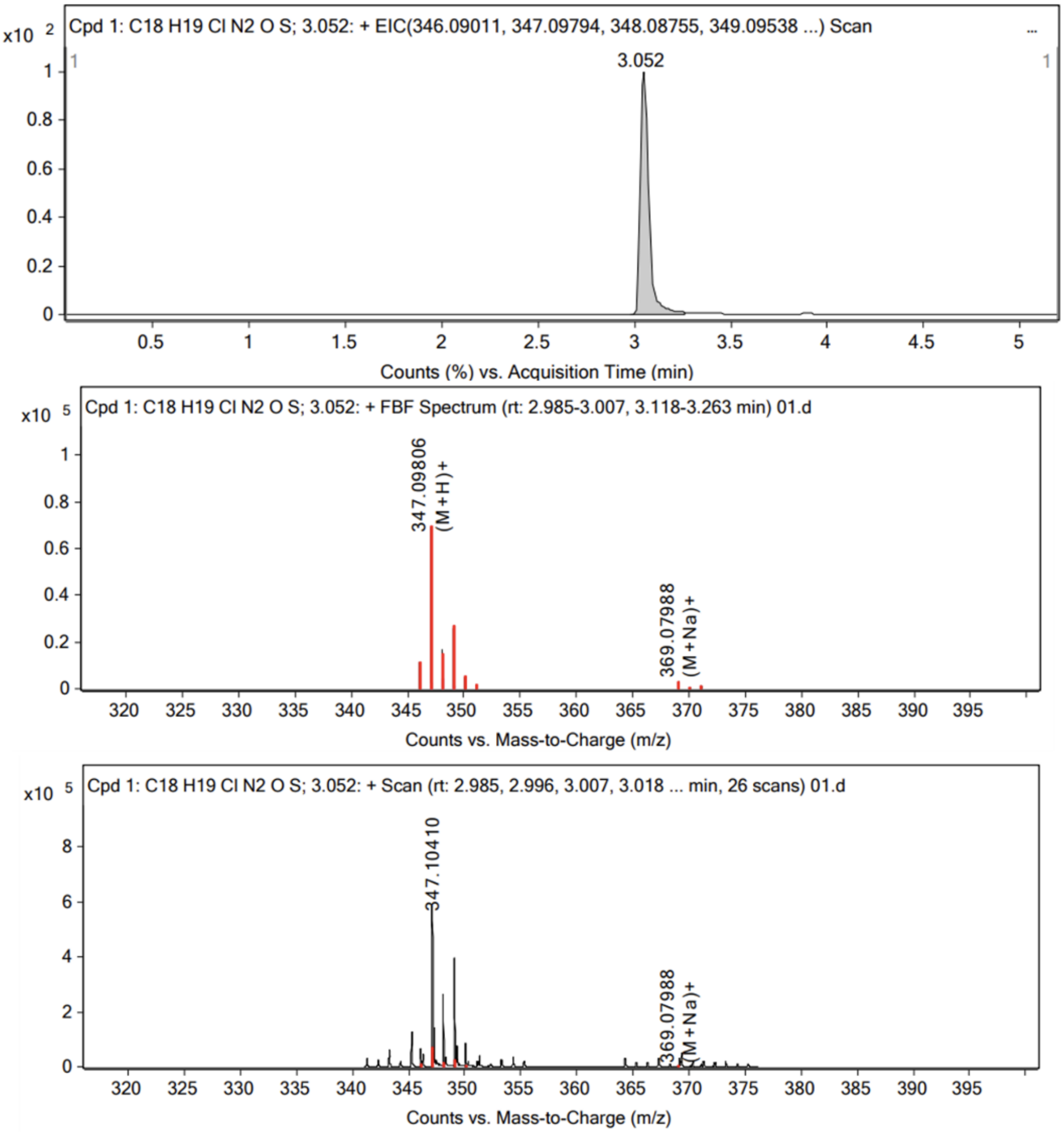

Supplementary Fig. 14c | SP11 HRMS quality control data

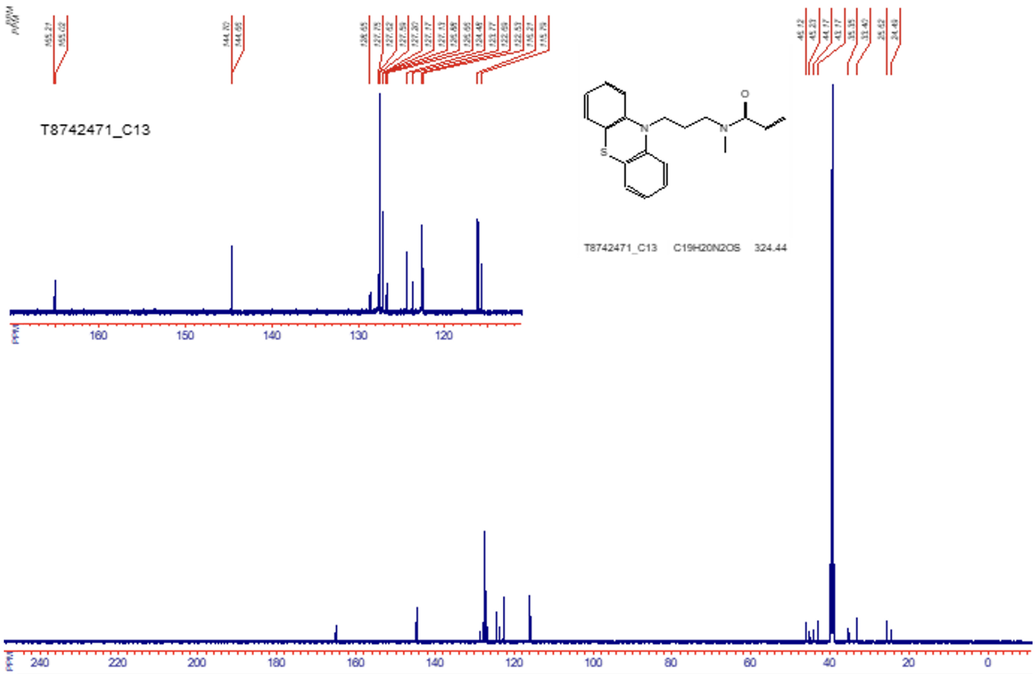

Supplementary Fig. 15a | SP22 ^{13}C NMR spectrum.

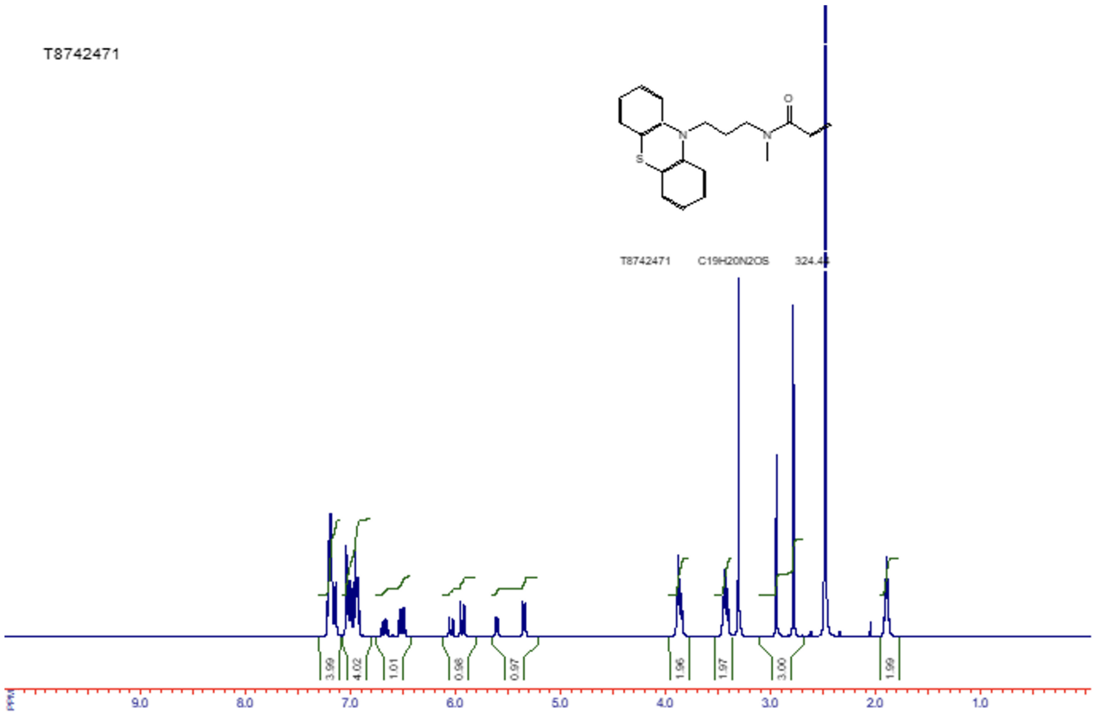

Supplementary Fig. 15b | SP22 ^{1}H NMR spectrum.

Compound Table

Label	Tgt Score	Mass Error (ppm)	Tgt Formula	Obs. RT	Ref. Mass	Obs. Mass
Cpd 1: C19 H20 N2 O S; 2.997	98.73	-3.99	C19 H20 N2 O S	2.997	324.12963	324.12834

Obs. *m/z*	Obs. RT	Obs. Mass	Tgt Formula	Tgt Mass	Tgt Mass Error (ppm)	RT Diff.	Find Cpds Algorithm
325.13679	2.997	324.12834	C19 H20 N2 O S	324.12963	-3.99	Find By Formula	

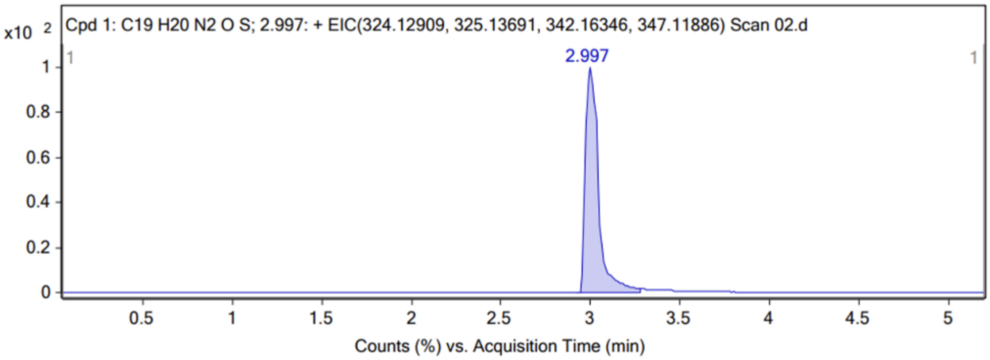

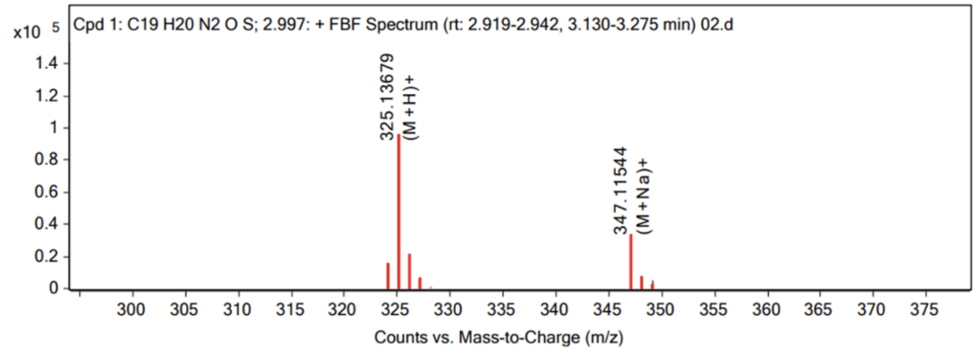

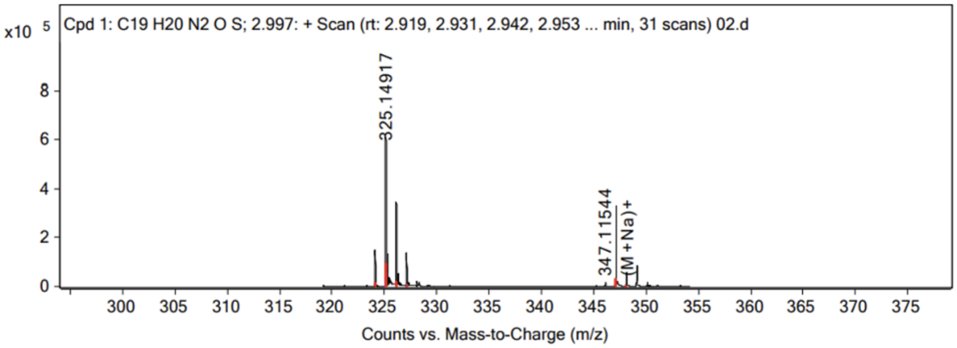

Supplementary Fig. 15c | SP22 HRMS quality control data

Small angle X-ray experiment and X-ray Crystallography

>WT Fis1
MEAVLNELVSVEDLLKFEKKFQSEKAAGSVSKSTQFEYAWCLVRSKYNDDIRKGIVLLEELLPK
GSKEEQRDYVFYLAVGNYRLKEYEKALKYVRGLLQTEPQNNQAKELERLIDKAMKKDGLLEVL
FQ

>Thr34Asp Fis1
MEAVLNELVSVEDLLKFEKKFQSEKAAGSVSKS**D**QFEYAWCLVRSKYNDDIRKGIVLLEELLPK
GSKEEQRDYVFYLAVGNYRLKEYEKALKYVRGLLQTEPQNNQAKELERLIDKAMKKDGLLEVL
FQ

>Tyr38Glu Fis1
MEAVLNELVSVEDLLKFEKKFQSEKAAGSVSKSTQFE**E**AWCLVRSKYNDDIRKGIVLLEELLPK
GSKEEQRDYVFYLAVGNYRLKEYEKALKYVRGLLQTEPQNNQAKELERLIDKAMKKDGLLEVL
FQ

CPM fluorescence assay and His-bead immobilization assay

>WT Fis1
MEAVLNELVSVEDLLKFEKKFQSEKAAGSVSKSTQFEYAWCLVRSKYNDDIRKGIVLLEELLPK
GSKEEQRDYVFYLAVGNYRLKEYEKALKYVRGLLQTEPQNNQAKELERLIDKAMKKDGLLEVL
FQGPHHHHHH

>Thr34Asp Fis1
MEAVLNELVSVEDLLKFEKKFQSEKAAGSVSKS**D**QFEYAWCLVRSKYNDDIRKGIVLLEELLPK
GSKEEQRDYVFYLAVGNYRLKEYEKALKYVRGLLQTEPQNNQAKELERLIDKAMKKDGLLEVL
FQGPHHHHHH

>Tyr38Glu Fis1
MEAVLNELVSVEDLLKFEKKFQSEKAAGSVSKSTQFE**E**AWCLVRSKYNDDIRKGIVLLEELLPK
GSKEEQRDYVFYLAVGNYRLKEYEKALKYVRGLLQTEPQNNQAKELERLIDKAMKKDGLLEVL
FQGPHHHHHH

>Cys41Ser Fis1
MEAVLNELVSVEDLLKFEKKFQSEKAAGSVSKSTQFEYAW**S**LVRSKYNDDIRKGIVLLEELLPK
GSKEEQRDYVFYLAVGNYRLKEYEKALKYVRGLLQTEPQNNQAKELERLIDKAMKKDGLLEVL
FQGPHHHHHH

> Cys41Ser, Val56Cys Fis1
MEAVLNELVSVEDLLKFEKKFQSEKAAGSVSKSTQFEYAW**S**LVRSKYNDDIRKGI**C**LLEELLPK
GSKEEQRDYVFYLAVGNYRLKEYEKALKYVRGLLQTEPQNNQAKELERLIDKAMKKDGLLEVL
FQGPHHHHHH

Supplementary Fig. 16 | Fis1 constructs used in experiments.

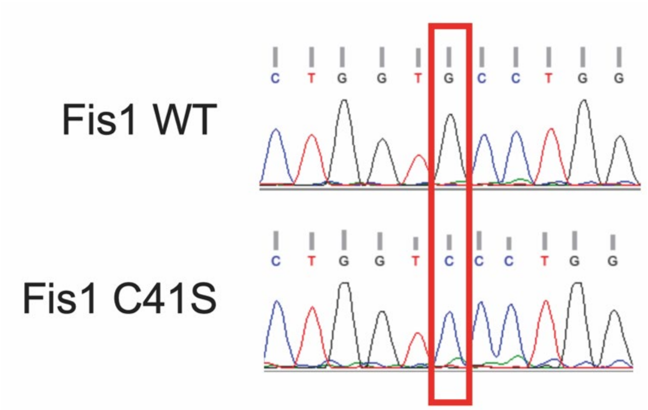

Gene Name	FIS1
Transcript ID	ENST00000223136.5
Guide RNA Sequence	AGUACGCCUGGUGCCUGGUG
Guide RNA Cut Location	Chr7: 101,244,059
Donor Sequence	GAAGGCAGCAGGCTCGGTGTCCAAGAGCACGCAGTTTGAGTAC GCCTGGTCCCTGGTGCGCAGCAAGTACAATGATGACATCCGTA AAGGCATCGTGCTGC
PCR Primers	FOR Primer (5'–3'): AATGGTGGGCGGTAAGTAGC<br>REV Primer (5'–3'): GTGCCAGATACCGAGACCTG
Sequencing Primer	Reverse
GC Enhancer Used	Yes

Supplementary Fig. 17 | Sequencing results and gene editing information for WT and Fis1 C41S Be(2)-M17 cells.

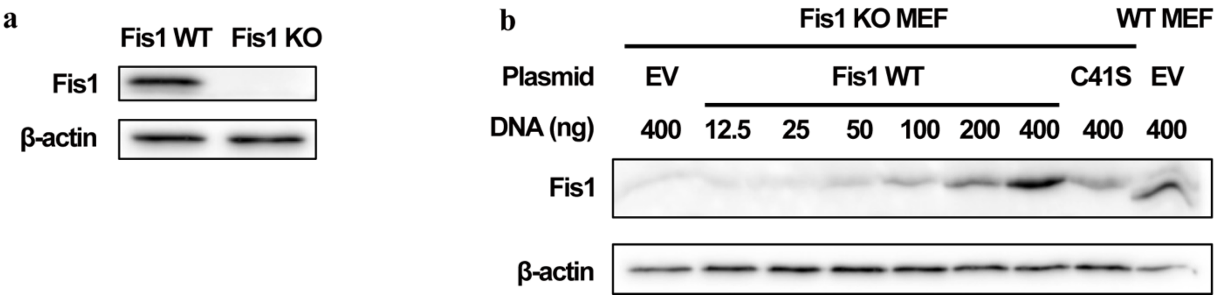

Supplementary Fig. 18 | Validation of Fis1 KO MEFs and titration of Fis1 transfection. a, Total proteins from WT MEFs and Fis1 KO MEFs were analyzed using immunoblotting. **b,** Fis1 KO MEFs and

WT MEFs were transfected with the indicated vectors for 24 h. Total proteins from the cells were analyzed using immunoblotting. EV, empty vector; WT, wild-type; KO, knock-out.

Fis1 WT
AAGCTTGCCACCATGGAAGCCGTGCTGAACGAGCTGGTTTCTGTGGAAGATCTGCTGAAGTT
CGAGAAGAAGTTTCAGAGCGAGAAAGCCGCTGGAAGCGTGTCCAAGTCCACCCAGTTCGAG
TACGCCTGGTGCCTGGTGCGGAGCAAGTACAACGACGACATCAGAAAGGGCATCGTGCTGC
TGGAAGAGCTGCTCCCCAAGGGCAGCAAGGAAGAGCAGAGAGATTACGTGTTCTACCTGGC
TGTGGGAAATTACAGACTGAAGGAATACGAGAAGGCCCTGAAATATGTGCGGGGCCTGCTG
CAAACAGAGCCTCAGAACAACCAGGCCAAGGAGCTGGAAAGACTGATCGACAAGGCCATG
AAAAAGGACGGCCTGGTCGGAATGGCCATCGTGGGCGGCATGGCTCTGGGCGTGGCCGGCC
TGGCCGGCCTTATCGGCCTGGCCGTGTCTAAAAGCAAGAGCGGGCCC

Fis1 C41S
AAGCTTGCCACCATGGAAGCCGTGCTGAACGAGCTGGTTTCTGTGGAAGATCTGCTGAAGTT
CGAGAAGAAGTTTCAGAGCGAGAAAGCCGCTGGAAGCGTGTCCAAGTCCACCCAGTTCGAG
TACGCCTGGAGCCTGGTGCGGAGCAAGTACAACGACGACATCAGAAAGGGCATCGTGCTGC
TGGAAGAGCTGCTCCCCAAGGGCAGCAAGGAAGAGCAGAGAGATTACGTGTTCTACCTGGC
TGTGGGAAATTACAGACTGAAGGAATACGAGAAGGCCCTGAAATATGTGCGGGGCCTGCTG
CAAACAGAGCCTCAGAACAACCAGGCCAAGGAGCTGGAAAGACTGATCGACAAGGCCATG
AAAAAGGACGGCCTGGTCGGAATGGCCATCGTGGGCGGCATGGCTCTGGGCGTGGCCGGCC
TGGCCGGCCTTATCGGCCTGGCCGTGTCTAAAAGCAAGAGCGGGCCC

Supplementary Fig. 19 | Sequences for Fis1 insert.

>sp|Q9Y3D6|FIS1_HUMAN Mitochondrial fission 1 protein [*Homo sapiens*]
MEAVLNELVSVEDLLKFEKKFQSEKAAGSVSKSTQFEYAWCLVRSKYNDDIRKGIVLLEE
LLPKGSKEEQRDYVFYLAVGNYRLKEYEKALKYVRGLLQTEPQNNQAKELERLIDKAMKK
DGLVGMAIVGGMALGVAGLAGLIGLAVSKSKS

>XP_039602965.1 mitochondrial fission 1 protein [*Polypterus senegalus*]
MEAVVNEVVSPEDLLKFEKKYNGEFTKGQVSKGTQFEYAWCLIRSKYSPDIKKGILLLEE
LVPKGTKDELRDYLFYLAVANYRLKEYEKGLKYIRTLLKNEPNNNQALELEKLINNAMKK
DGLVGMAIVGGIGLGVAGLAGLIGLAVAKSKS

>XP_051900758.1 mitochondrial fission 1 protein [*Pristis pectinata*]
MEAIVNETVSSEDLLKFEKKYNLELKQGSLSRATQFEYAWCLIRSKYSGDIVKGVAILEE
LYPDASKEEQRDYIFYLAVGNYRLKEYEKALKFIRTMLKNEPRNQQCLELEKLINKKMQR
DGLLGMAIVGGAIVGLAGMAGLIGLAVSKGKS

>XP_053321064.1 mitochondrial fission 1 protein [*Spea bombifrons*]
METVLSDLVAVEDLVRFEKKYLAELSAGALSKSTQFEYAWCLIRSKYSDDIRKGAVLLEE
LLPKGSKEEQRDYLFYLSVAHYRLKEYEKALRYIRTLLSAEPNNSQAKEVEKVIEKTMQK
DGLVGMAIVGGMALGVAGLAGLIGLAISKSK

>XP_032836180.1 mitochondrial fission 1 protein isoform X2 [*Petromyzon marinus*]
MEAILNDFISAEDLMKFEKKYNAEMAKGPLSPATQFEYSWCLVRSRHSEDVKRGVRLLED
LCHNAKGDELRDYLFYISIGYYRLKDYEKALKFVKTLLRKEPTNQQALQLEKLINKAMQK
DGLVGMAIVGGVVVGLAGLAALAIHKGTR

>XP_030398511.1 mitochondrial fission 1 protein [*Gopherus evgoodei*]
MESVLSEVVAVEDLLRFEKKYNAELSGGTVSKGTQFEYAWCLVRSKYNDDIRKGIVLLED
LVPRGCKEEQRDYVFYLAVANYRLKEYEQALKYIRGLLKTEPTNTQALELEKLIDKAMQK
DGLVGMAIVGGMALGVAGLAGLIGLAISKSKS

Supplementary Fig. 20 | Sequences used for Fis1 alignment

Molecular Dynamics Simulations Checklist

**Reliability and reproducibility checklist for molecular dynamics simulations**<br>***All boxes must be marked YES by acceptance unless "Response not needed if No".**	**Yes**	**No**	**Response (Please state where this information can be found in the text)**
**1. Convergence of simulations and analysis**			
1a. Is an evaluation presented in the text to show that the property being measured has equilibrated in the simulations (*e.g.* time-course analysis)?	☒	☐	Time-series analyses in the manuscript indicate that the system has reached equilibrium.<br>In particular, to evaluate whether the solvent-accessible surface area (SASA) of Cys41 (Fig. 3b) and the secondary structural propensities of residues in the α1 helix are in equilibrium, time series data of these values are shown in Fig. 3b and Supplementary Fig. 1.

1b. Then, is it described in the text how simulations are split into equilibration and production runs and how much data were analyzed from production runs?	☒	☐	We have now provided these details in the "Methods". Specifically, we clarified how the simulations were split into equilibration and production runs, and we specified how much data from the production run were used for analysis.
1c. Are there at least 3 simulations per simulation condition with statistical analysis?	☒	☐	We performed 30 MD simulations of 1.2 μs for the WT-Fis1, the pThr34 and the pTyr38 (1.2 μs × 90 MD simulations in total), described in the "Methods" section.
1d. Is evidence provided in the text that the simulation results presented are independent of initial configuration?	☒	☐	We performed 30 independent MD simulations for each system by assigning different initial velocities using distinct random seeds under a Langevin thermostat (described in the "Methods" section), ensuring that our results do not depend on the initial configuration, as shown in Fig. 3b.
**2. Connection to experiments**			
2a. Are calculations provided that can connect to experiments (*e.g.* loss or gain in function from mutagenesis, binding assays, NMR chemical shifts, J-couplings, SAXS curves, interaction distances or FRET distances, structure factors, diffusion coefficients, bulk modulus and other mechanical properties, *etc.*)?	☒	☐	In the main text, we demonstrate clear links between the MD simulation results and experimental data in two sections. First, in "The α1 helix of Fis1 undergoes

			conformational change upon activation," we compare the results obtained by our simulations and those obtained by X-ray crystallography and small-angle X-ray scattering (SAXS). Additionally, in "Conformational change in α1-helix unfolding and Fis1 activation expose the one and only cysteine, Cys41, in Fis1," we relate the simulations to CPM fluorescence measurements.
**3. Method choice**			
3a. Do simulations contain membranes, membrane proteins, intrinsically disordered proteins, glycans, nucleic acids, polymers, or cryptic ligand binding?	☐	☒	Response not needed if **No**
3b. Is it described in the text whether the accuracy of the chosen model(s) is sufficient to address the question(s) under investigation (e.g. all-atom vs. coarse-grained models, fixed charge vs. polarizable force fields, implicit vs. explicit solvent or membrane, force field and water model, etc.)?	☒	☐	We selected an all-atom model to capture the detailed dynamics and phosphorylation effects on Fis1. We also discuss the validity of our force fields and modeling approach.
3c. Is the timescale of the event(s) under investigation beyond the brute-force MD simulation timescale in this study that enhanced sampling methods are needed?	☐	☒	
If **YES**, are the parameters and convergence criteria for the enhanced sampling method clearly stated?	☐	☐	
If **NO,** is the evidence provided in the text?	☒	☐	We observed Cys41 exposures occurring within the timescale of our conventional MD simulations, as evidenced by the time-series data shown in

				Fig. 3b and Supplementary Fig. 1. Therefore, enhanced sampling methods were not required for capturing these events.
**4. Code and reproducibility**				
4a. Is a table provided describing the system setup that includes simulation box dimensions, total number of atoms, total number of water molecules, salt concentration, lipid composition (number of molecules and type)?		☒	☐	We have included Supplementary Table 3 that details the system setup for the MD simulations.
4b. Is it described in the text what simulation and analysis software and which versions are used?		☒	☐	We specify the simulation and analysis software, along with their versions, in the "Methods" section.
4c. Are other parameters for the system setup described in the text, such as protonation state, type of structural restraints if applied, nonbonded cutoff, thermostat and barostat, etc.?		☒	☐	We describe all relevant parameters in the "Methods" section, including protonation states, any structural restraints used, the nonbonded cutoff, and the specifics of the thermostat and barostat.
4d. Are initial coordinate and simulation input files and a coordinate file of the final output provided as supplementary files or in a public repository?		☒	☐	We have made them publicly available through Google Drive link in the Source Data file.
4e. Is there custom code or custom force field parameters?		☐	☒	Response not needed if **No**
	If **YES**, are they provided as supplementary files or in a public repository?	☐	☐	
