# Supplementary material for: A hidden cysteine in Fis1 targeted to prevent excessive mitochondrial fission and dysfunction under oxidative stress
Source: Nat Commun. 2025 May 6;16:4187. doi: 10.1038/s41467-025-59434-6 (PMC12056058; doi:10.1038/s41467-025-59434-6)
Supplement: Supplementary file 2 — Description of Additional Supplementary Files [file 41467_2025_59434_MOESM2_ESM.docx]

**Description of Additional Supplementary Files**

**Supplementary Movie 1:** Dynamic nature of phosphorylated Fis1. One 1.2μs-MD simulation of pY38-Fis1 is shown. The α1 helix is colored orange, and pY38 and C41 are highlighted in licorice style.
